# Peritoneal Tuberculosis After Robot-Assisted Laparoscopic Prostatectomy with Extended Lymph Node Dissection

**DOI:** 10.1089/cren.2018.0018

**Published:** 2018-04-01

**Authors:** Suruga Saito, Katsuhiro Ito, Keiyu Matsumoto, Motofumi Tajima, Takayuki Goto, Haruki Ito, Yumi Manabe, Mutsuki Mishina, Hiroshi Okuno

**Affiliations:** Department of Urology, National Hospital Organization Kyoto Medical Center, Kyoto, Japan.

**Keywords:** complication, extended lymph node dissection, peritoneal tuberculosis, robot-assisted laparoscopic prostatectomy

## Abstract

***Background:*** Peritoneal tuberculosis (TB) is a relatively uncommon presentation of extrapulmonary TB. Early diagnosis of peritoneal TB is difficult because of its nonspecific clinical manifestation such as abdominal pain, fever, or ascites. Especially early after surgery of abdomen or pelvis, these symptoms can be misdiagnosed as septic peritonitis. There are few reports of peritoneal TB as a postoperative complication of laparoscopic surgery. Here, we describe a first case of peritoneal TB after robot-assisted laparoscopic prostatectomy (RALP) with extended lymph node dissection.

***Case Presentation:*** A 78-year-old man presented 25 days after this surgery with fever and abdominal distension. Ultrasonography and computed tomography (CT) revealed massive abdominal ascites. Ascites sample was cloudy, with increased white blood cells and normal creatinine level. No anastomotic leak was found. Bacterial infection of a lymphocele was considered, and cefmetazole 2 g/day for 3 days was prescribed. Despite antibacterial therapy, fever persisted. Polymerase chain reaction testing of ascitic fluid was positive for *Mycobacterium tuberculosis*. The patient was effectively treated with anti-TB therapy.

***Conclusion:*** This is the first report of peritoneal TB as a postoperative complication of RALP with extended lymph node dissection. His preoperative chest CT showed granular shadows in left upper lung, indicating his old asymptomatic TB infection. Flare-up of TB can happen even after robot-assisted laparoscopic surgery, which is minimally invasive. Peritoneal TB must be considered especially when there is unexplained ascites unresponsive to antibiotics.

## Introduction

Peritoneal tuberculosis (TB) is a relatively uncommon presentation of extrapulmonary TB. It is difficult to diagnose rapidly and accurately because of its nonspecific symptoms (e.g., abdominal distension, pain, and fever) and the difficulty of isolating *Mycobacterium tuberculosis*. Early recognition of peritoneal TB is important because treatment delay is associated with high mortality rates.^1^ We describe a case of peritoneal TB after robot-assisted laparoscopic prostatectomy (RALP) with extended lymph node dissection.

## Presentation of Case

A 78-year-old man with no significant history was found to have prostate cancer stage cT2cN0M0, Gleason score of 4 + 5 with a serum prostate-specific antigen value of 8.05 ng/cc in June 2016. He underwent RALP with extended lymph node dissection. The operation time was 339 minutes and bleeding volume was 150 cc. The Foley catheters were removed on postoperative day 6, according to the usual protocol. No urinary leakage from the anastomosis sites was observed. He was discharged without event on postoperative day 14. Pathologic diagnosis was pT3b, Gleason score of 4 + 5, and positive resection margin. Lymph node metastasis was not detected.

**FIG. 1.**
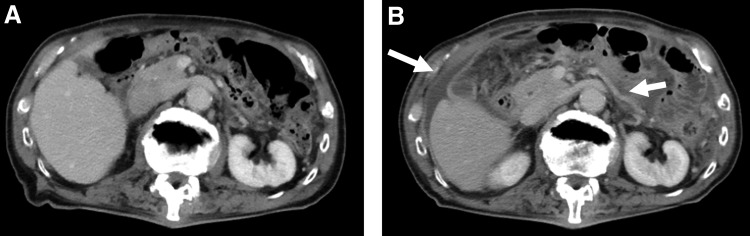
CT findings in the abdomen: **(A)** preoperative and **(B)** postoperative. *Arrows* point to the thickened peritoneum.

**FIG. 2.**
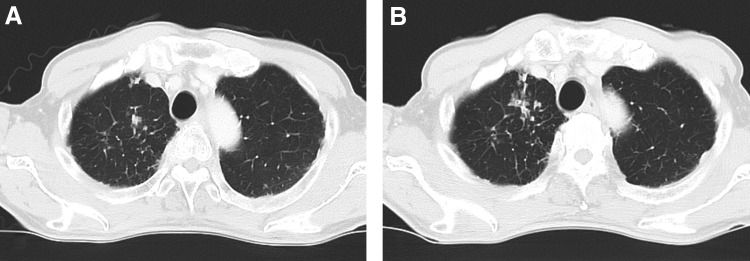
CT of the left upper lung: **(A)** preoperative and **(B)** postoperative. The granular shadows and a mucous plug indicate an old TB infection. TB, tuberculosis.

On postoperative day 25, he presented with fever and general fatigue. Physical examination showed body temperature of 38.2°C, regular pulse (84/min), blood pressure 145/84 mm Hg, and a distended abdomen. Initial laboratory findings were a normal white blood cell (WBC) count and increased C-reactive protein level (11.2 mg/dL). Liver and renal functions were normal. Computed tomography (CT) showed massive ascites and extensive thickened peritoneum (Fig. 1). CT of lungs showed slightly worsening granular shadows and a mucous plug in the left upper lobe (Fig. 2). Initial ascites sample was cloudy, with increased WBCs and normal creatinine level, precluding urinary leakage. Bacterial infection of a lymphocele was considered and cefmetazole 2 g/day for 3 days was prescribed but was ineffective. Blood, urine, sputum, and ascites cultures were negative.

**FIG. 3.**
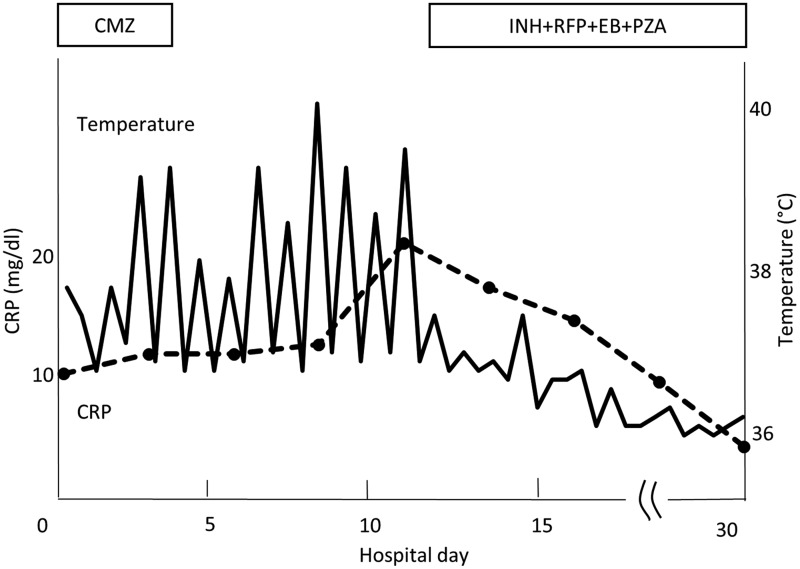
Clinical course of the patient's admission. The *dotted line* indicates his body temperature. The *solid line* indicates the CRP levels. CMZ, cefmetazole; CRP, C-reactive protein; EB, ethambutol; INH, isoniazid; PZA, pyrazinamide; RFP, rifampicin.

Repeat abdominocentesis revealed 2900 WBCs/mm^3^ (87% lymphocytes) and adenosine deaminase of 140 IU/L (normal 0–33 IU/L). Ziehl–Neelsen staining and initial polymerase chain reaction (PCR) for *M. tuberculosis* were negative. Interferon-γ release assay was indeterminate. Abdominocentesis was performed repeatedly, attempting to diagnose peritoneal TB. The fourth ascites sample was positive. The cultures and PCR of urine, sputum, and gastric lavage for TB were negative. The patient was found to have peritoneal TB. Anti-TB therapy with isoniazid (INH) (300 mg/day), rifampicin (RFP) (450 mg/day), ethambutol (EB) (1000 mg/day), and pyrazinamide (PZA) (1200 mg/day) was initiated. His temperature dropped immediately, and his general condition improved (Fig. 3). After the initial 2-month phase, INH and RFP were given for another 4 months. Three months after oral treatment completion, there was no recurrence of infection or ascites.

## Discussion

TB is an infectious disease caused by *M. tuberculosis*. Although new TB infections are rare in developed countries, Japan still has an intermediate TB incidence, with increasing reports of extrapulmonary TB. Although the peritoneum is an uncommon site, cirrhosis, alcoholic liver disease, and acquired immune deficiency syndrome are known risk factors for peritoneal TB.^2^ Since only a few reports of peritoneal TB as surgical complication have been published, it has not been clarified which kinds of surgery or perioperative factors are risk of infection.

TB can infect the peritoneum by various mechanisms. Hematogenous spread of tubercles from a primary pulmonary focus is most common.^2^ Dissemination from adjacent organs (e.g., intestine and genitourinary organs) occurs, but is rare.^3^ Most TB-infected humans have been living with it without progression, although the tubercular bacilli may be disseminated anywhere in the body. Although this patient had no apparent history of TB, his wife had a history of pulmonary TB, and his chest CT indicates old TB infection. Thus, he may have been infected without symptoms. Subsequently, TB may have spread from lung to peritoneum hematogenously. Precise history taking and screening for active TB infection should be necessary before surgery when preoperative findings show possible past TB infection like our case. Latent tubercular bacilli can reactivate when the host's immune system weakens, possibly spreading to the peritoneum. There is no report of peritoneal TB after RALP. However, postoperative flare-up of TB is not an uncommon clinical entity. It is conceivable that flare-up of TB happens after even RALP, which is a minimally invasive surgery. Lymphocele because of extended lymphadenectomy and a weakened peritoneal immune system because of laparoscopic surgery might have made him vulnerable to latent TB.

TB dissemination from the genitourinary tract or dissected lymph nodes has been recognized. Our patient's dissected prostate and lymph nodes were re-examined, but there was no histologic evidence of genitourinary tubercular infection. Urine cultures and PCR were also negative for TB, suggesting that his infection had not originated in genitourinary organs or lymph nodes.

Peritoneal TB diagnosis is complicated by the nonspecific symptoms and limited diagnostic tests. Abdominal pain, fever, weight loss, and ascites are common symptoms,^2^ but are also common in bacterial peritonitis. The postoperative condition makes it difficult to diagnose peritoneal TB. Ascites culturing has low sensitivity (30%)^2^ and thus requires retesting to obtain positive results, leading to delayed diagnoses. Ascites adenosine deaminase is a useful diagnostic marker for peritoneal TB. The diagnostic sensitivity is >90% when the cutoff is 30 IU/L.^2^ PCR of ascitic fluid for *M. tuberculosis* has high specificity but low sensitivity. To consider the low sensitivity and high cost of PCR, routine use of PCR is difficult. However, PCR can identify TB more quickly than culture. Repeat PCR may contribute to the diagnosis of high probability of peritoneal TB for patients or those who need rapid diagnosis. If these tests are negative, diagnostic laparoscopy and targeted biopsy would be necessary for accurate diagnosis.^2^

The dose and schedule of anti-TB drugs are the same for pulmonary and peritoneal TB. The 2-month initial phase comprises daily INH, RFP, PZA, and EB, followed by INH and RFP for another 4 months.^4^

## Conclusion

Physicians who perform RALP should be aware that peritoneal TB could develop after surgery with lymph node dissection. TB must be considered especially when there is unexplained ascites unresponsive to antibiotics.

## Abbreviations Used

CMZ: cefmetazole
CRP: C-reactive protein
CT: computed tomography
EB: ethambutol
INH: isoniazid
PCR: polymerase chain reaction
PZA: pyrazinamide
RALP: robot-assisted laparoscopic prostatectomy
RFP: rifampicin
TB: tuberculosis
WBC: white blood cell
